# Towards a Closed-Loop Bioengineering Framework for Immersive VR-Based Telerehabilitation Integrating Wearable Biosensing and Adaptive Feedback

**DOI:** 10.3390/bioengineering13040439

**Published:** 2026-04-09

**Authors:** Gaia Roccaforte, Arianna Sinardi, Sofia Ruello, Carmela Lipari, Flavio Corpina, Antonio Epifanio, Anna Isgrò, Francesco Davide Russo, Alfio Puglisi, Giovanni Pioggia, Flavia Marino

**Affiliations:** 1 Department of Biomedical, Dental and Morphological and Functional Imaging Sciences, University of Messina, 98122 Messina, Italy; 2 Institute for Biomedical Research and Innovation (IRIB), National Research Council of Italy (CNR), 98164 Messina, Italy; 3 Foresight Consulting, 98122 Messina, Italy; 4 Euro-Mediterranean Institute of Science and Technology, 90139 Palermo, Italy; 5 Department of Economics, University of Messina, 98122 Messina, Italy; 6 Research Unit of Artificial Intelligence and Computer Systems, Department of Engineering, Università Campus Bio-Medico di Roma, 00128 Rome, Italy; 7 Società Servizi Riabilitativi, 98125 Messina, Italy

**Keywords:** telerehabilitation, virtual reality, wearable devices, gamification, neurological rehabilitation, home-based therapy, adaptive feedback, remote monitoring, patient engagement, data-driven rehabilitation

## Abstract

Telerehabilitation—the remote delivery of rehabilitation services—is undergoing a paradigm shift with the convergence of immersive virtual reality (VR) and wearable biosensor technologies. This perspective article outlines a vision for home-based motor and cognitive rehabilitation that is engaging, personalized, and data-driven. We describe how immersive VR environments (for example, simulations of home settings or supermarkets) coupled with wearable sensors can address current challenges in rehabilitation by increasing patient motivation, enabling real-time biofeedback, and supporting remote clinician supervision. Gamification mechanisms and rich sensory feedback in VR are highlighted as key strategies to enhance user engagement and adherence to therapy. We discuss conceptual innovations such as multi-sensor data integration, dynamic difficulty adaptation, and AI-driven personalization of exercises, derived from recent research and our development experience, and consider their potential benefits for patients with neuro-cognitive-motor impairments (e.g., stroke, Parkinson’s disease, and multiple sclerosis). Implementation scenarios for home-based therapy are presented, emphasizing scalability, standardized digital metrics for monitoring progress, and seamless involvement of clinicians via telehealth platforms. We also critically examine the current limitations of VR and telehealth rehabilitation and how an integrative model could overcome these barriers. More specifically, this perspective defines the engineering requirements of a closed-loop VR-based telerehabilitation framework, including multimodal data synchronization, calibration, signal-quality management, interpretable adaptive control, digital biomarker validation, and practical strategies to improve accessibility, privacy, and scalability in home-based neurological rehabilitation.

## 1. Introduction

Rehabilitation is increasingly embracing digital health innovations to expand access and improve outcomes. Telerehabilitation (TR), defined as the delivery of rehabilitation assessment and therapy via telecommunication technologies, has emerged as a viable alternative or complement to in-person care [1]. Especially in the wake of recent global challenges and an aging population, TR offers a means to continue therapy beyond hospital walls, reducing travel burdens and reaching patients who cannot easily attend clinics. Studies have shown that remote rehabilitation can yield functional outcomes comparable to face-to-face therapy for conditions like stroke, including improvements in balance and activities of daily living, while greatly enhancing accessibility [1]. TR can help maintain continuity of care during critical recovery periods while reducing geographic, mobility and logistical barriers to rehabilitation [1]. Indeed, recent protocol studies have started to evaluate integrated VR-based telerehabilitation platforms in older adults, focusing on usability and acceptability [2]. However, conventional telerehabilitation approaches (such as video-call supervised exercises) face limitations in engagement and feedback, potentially leading to patient boredom and suboptimal adherence.

In parallel, technological advances in immersive virtual reality (VR) and wearable biosensors are creating new opportunities to transform rehabilitation [3]. In recent years there is growing interest in leveraging immersive VR environments, such as computer-generated 3D worlds experienced via head-mounted displays, to simulate functional real-life tasks in a safe, controlled setting [4,5]. VR-based rehabilitation builds on well-established neurorehabilitation principles: it enables repetitive, task-specific practice with real-time feedback, which can drive neuroplasticity and motor learning more effectively than conventional exercises [4,5]. Immersive VR can be adjusted to individual patient needs and skill levels, increasing motivation and engagement compared to one-size-fits-all therapy [4,5]. At the same time, wearable sensors (such as inertial motion units (IMUs), pressure-sensing insoles, heart-rate monitors, etc.) allow continuous, objective monitoring of a patient’s movements and physiological responses during exercise. The convergence of these technologies promises an integrative telerehabilitation model: patients interact with gamified VR therapy scenarios at home while wearable biosensors capture quantitative data (e.g., kinematics and vital signs), all connected to clinicians through a network. This model shifts rehabilitation toward a home-based but data-driven paradigm, where therapy is both engaging and closely monitored.

This perspective article aims to move beyond a generic discussion of potential and to define a clinically grounded bioengineering framework for immersive VR-based telerehabilitation integrated with wearable biosensing. Specifically, the article addresses four research questions: (i) which engineering components are required to build a closed-loop rehabilitation system that synchronizes immersive VR events with multimodal wearable data; (ii) which digital biomarkers can be extracted from motor, cognitive, and physiological streams to support adaptive rehabilitation; (iii) how these biomarkers can be linked to clinical scales and used to personalize training while preserving interpretability and clinical oversight; and (iv) which technical and organizational strategies are necessary to make home-based deployment feasible, scalable, and accessible for neurologically impaired patients. Accordingly, the objectives of this article are fourfold: first, to define the functional architecture of an immersive VR-biosensor telerehabilitation platform; second, to identify the main engineering and clinical implementation challenges, including multimodal data synchronization, calibration, interoperability, and safety monitoring; third, to propose an interpretable AI-enabled adaptation framework based on digital biomarkers derived from task performance and physiological monitoring; and fourth, to outline practical strategies for home-based implementation, including low-burden hardware configurations, caregiver support, remote clinical supervision, and staged deployment models. The article therefore frames immersive telerehabilitation not simply as a promising concept, but as a translational bioengineering problem requiring explicit technical, clinical, and organizational design choices.

## 2. Current Challenges in Telerehabilitation

This section does not aim to provide an exhaustive review but rather to highlight key recurring challenges that motivate the need for a new telerehabilitation paradigm. Despite promising advances, today’s telerehabilitation approaches face several challenges that limit their effectiveness and adoption. One major issue is patient engagement and adherence. Traditional rehabilitation exercises, especially when performed remotely with minimal interactivity, can be repetitive and monotonous, leading to patient disengagement over time [6]. Low motivation and boredom contribute to poor adherence to exercise regimens, undermining the potential benefits of therapy. This is compounded by the fact that rehabilitation often requires long-term, intensive training—a regimen that many patients struggle to maintain without consistent encouragement or enjoyment. Moreover, conventional telerehab (e.g., supervised video exercises or teleconsultations) provides limited sensory feedback and lacks the interactive context that can make exercises meaningful, further reducing patient interest. In sum, sustaining active participation is a known hurdle: high dropout rates or suboptimal exercise frequency are reported when therapy is not engaging. Enhancing motivation and adherence is therefore a critical need in remote rehabilitation programs.

Another challenge lies in providing real-time feedback and objective monitoring during at-home therapy. In clinical settings, therapists closely observe patient movements, form, and effort, giving instant corrections or encouragement. Remotely, such detailed monitoring is difficult with simple video calls or periodic progress check-ins. Important aspects of performance, like precise motion kinematics, balance shifts, reaction times and physiological exertion, may go unquantified. The lack of high-quality data on patient performance makes it harder to tailor therapy to the individual or to detect subtle improvements. Furthermore, ensuring patient safety in unsupervised environments is a concern; without proper monitoring, signs of overexertion or cardiovascular stress might be missed. While some telerehab systems have begun to incorporate sensors for remote patient monitoring, integration is often limited (for example, a single wearable or basic activity tracker) and not deeply embedded into the therapy feedback loop.

Accessibility and scalability issues also persist. Telerehabilitation was developed in part to improve access for patients in remote or underserved areas and to reduce healthcare costs [7]. However, disparities remain in who can effectively use current telerehab solutions. Many platforms require reliable high-speed internet and a certain level of technical literacy, potentially excluding older adults or those of lower socioeconomic status who may lack these resources [8]. Additionally, healthcare providers and systems have been slow in some cases to adopt telerehab technology, citing barriers such as lack of training and interoperability issues between systems [7]. Early reviews noted that many telerehab tools were not interoperable with electronic health records or across vendors, impeding a seamless flow of data and integration into clinical practice [7]. While the COVID-19 pandemic accelerated the uptake of telehealth, there is still work to be done to make telerehabilitation widely scalable and standardized across institutions. Standardizing outcome measurement parameters is important: different studies and programs use different scales and measurements to assess progress, making it difficult to compare results or determine overall effectiveness [1]. A recent scoping review of post-stroke telerehab noted a broad range of outcome measures used across trials, highlighting the need for consensus on the most appropriate metrics and follow-up assessments [1].

Importantly, the current evidence base for advanced telerehab technologies (like VR and AI-driven systems) is still maturing. Many studies conducted to date have small sample sizes, short intervention durations, or heterogeneous designs [4,9]. This heterogeneity and the paucity of large randomized trials make it challenging to draw definitive conclusions about long-term effectiveness. For instance, an umbrella review of VR-based physiotherapy in PD found moderate benefits for daily functioning but also identified significant variability in interventions and difficulties in blinding, which complicate interpretation of results [4]. Similarly, systematic reviews of sensor-based or VR rehab often report that while short-term improvements are observed, the strength of evidence is limited by methodological shortcomings and lack of long-term follow-up [9]. These findings highlight the need for more robust clinical validation and integrative frameworks capable of unifying emerging modalities. In summary, current telerehabilitation faces a convergence of challenges: keeping patients engaged and adherent, monitoring them comprehensively and safely at home, making solutions accessible and standardized, and bolstering the evidence base. The following sections discuss how an integrative approach using immersive VR and wearable biosensors can directly target these issues, representing a new paradigm in remote rehabilitation.

### 2.1. Toward a New Paradigm: Immersive VR with Integrated Biosensing

To overcome the aforementioned challenges, we propose a paradigm shift in telerehabilitation centered on the integration of immersive virtual reality environments with wearable biosensor feedback. This approach creates a highly interactive therapeutic experience at home that closely replicates in-person rehabilitation in its ability to engage patients and monitor their progress, potentially complementing and, in specific contexts, enhancing rehabilitation methods. In an immersive VR-based telerehab session, the patient wears a VR headset that places them in a simulated real-life scenario relevant to therapy goals (for example, navigating a virtual supermarket, cooking in a virtual kitchen, or walking in a simulated street). These environments provide a safe and controlled yet realistic context for patients to practice functional tasks involving both motor and cognitive skills [4]. Crucially, VR allows tasks to be designed in a game-like format with clear objectives, immediate feedback, and an adjustable difficulty level. The patient’s movements are tracked in real time: contemporary VR systems can capture head and hand motions natively, and additional wearable motion sensors can be attached to limbs if needed for finer movement tracking. As a result, every reach, grip, or step the patient performs in the virtual environment can be recorded and assessed quantitatively.

Meanwhile, wearable biosensors monitor physiological and performance metrics to enrich the therapy data. Small wireless devices can collect motion data (joint angles, limb acceleration), muscle activity (via surface EMG), and vital signs like heart rate or respiration. Integrating these sensors with the VR system enables a continuous stream of multimodal data during exercise. For example, inertial measurement units (IMUs) on the lower limbs or torso can provide precise gait and balance information to complement the VR avatar’s movements [3,9]. A heart-rate sensor or smartwatch can track cardiovascular response, ensuring the exercise intensity stays within safe ranges and providing an indicator of exertion or stress. All these data are synchronized with the virtual task events, meaning that one can correlate how the patient’s body is responding at each moment of the therapy scenario. The combined system effectively creates a closed-loop rehabilitation platform: the patient’s actions in VR yield immediate feedback (visual, auditory, or haptic) and are simultaneously measured by sensors, and this information can then be used to adjust the therapy in real time or guide clinical decisions.

Immersive VR offers several exciting advantages for rehabilitation. First, it can provide real-time augmented feedback that is difficult to achieve otherwise. As the patient performs a task, the system can give instant visual cues (for instance, highlighting the target object to grab or displaying a balance gauge), auditory signals (success chimes or corrective prompts), or even haptic vibrations, thereby reinforcing correct performance or signaling errors. This kind of immediate knowledge of results is known to enhance motor learning and skill acquisition. Second, VR can present adaptive sensory and cognitive challenges that evolve with the patient’s improvement. In a virtual supermarket scenario, for example, early sessions might involve a nearly empty, quiet store with simple tasks (few items to pick, minimal distractions). As the patient’s cognitive-motor abilities improve, the scenario can gradually introduce more items on the shelves, background noise or crowds, time limits, or dual-task conditions (e.g., remembering a shopping list while selecting items). This gradual exposure to increasing difficulty is designed to continuously challenge the patient just enough to stay in the “learning zone”, in line with the concept of maintaining flow by balancing challenge and skill level. Indeed, VR systems can implement level progression algorithms: many immersive rehab exercises are organized in levels of rising complexity, where achieving a certain score or proficiency unlocks the next level. This ensures the therapy remains neither too easy (which could cause disengagement) nor too hard (which could cause frustration), but rather dynamically tuned to the patient’s current capabilities.

Equally important, the VR-sensor paradigm supports integrated motor and cognitive rehabilitation. Traditional physical therapy often focuses on isolated motor skills, while cognitive training might be done separately. In daily life, however, people must coordinate body and mind simultaneously, for example, walking while talking or planning a sequence of actions while executing them. Immersive VR is uniquely suited to practice such integrated tasks. Scenarios can be designed to incorporate cognitive exercises (memory, attention, problem-solving) into physical movements. The earlier supermarket example illustrates this: the patient must physically search and grab items (motor coordination) based on memory of a list or recognition of specific features (cognitive processing) under a time constraint. This holistic training could lead to more transfer of skills to real-world function, as it mirrors real activities more closely than rote exercises. Research suggests that adding cognitive or dual-task components in VR rehabilitation for neurological patients can improve executive function and lead to more sustained functional gains. For instance, in Parkinson’s disease, combining immersive game-like exercises with cognitive challenges improved not only motor outcomes but also resulted in longer-term maintenance of mobility gains, especially under dual-task conditions, compared to conventional therapy [4]. By engaging sensory-motor integration and higher-level cognition together, VR-based rehab may better address the multidimensional deficits seen in stroke, PD, MS and similar conditions.

Furthermore, an immersive VR approach can tackle psychosocial aspects of rehabilitation. Patients undergoing long recoveries often experience isolation or reduced social interaction, which can negatively impact mental health and motivation. VR can help alleviate this by enabling virtual social participation and support. Multi-user VR environments allow patients to engage in therapy jointly or share experiences; for example, a therapist’s avatar could join the patient’s virtual environment for a guided session. Such features introduce a social element that traditional home exercise lacks. Even in single-player modes, VR can simulate social contexts (like navigating a crowded public space or interacting with a virtual shopkeeper) that practice social cognition. A narrative review on VR in Parkinson’s noted that VR’s immersive, interactive features, including virtual group activities or environments mimicking social interactions, may help reduce feelings of loneliness and improve emotional well-being in addition to physical benefits [4]. This all-encompassing approach, addressing physical, cognitive, and psychosocial domains, is particularly valuable for neurodegenerative diseases where depression and social withdrawal are common [4]. In summary, integrating VR with biosensors creates a versatile telerehab platform: it immerses patients in meaningful, multi-faceted training scenarios; provides real-time, rich feedback; quantifies performance and physiology; and adapts to user needs. We next examine how gamification strategies build upon this foundation to further boost engagement and adherence.

A central engineering requirement of this paradigm is the synchronization of heterogeneous data streams generated by the VR engine, wearable sensors, and clinician-side monitoring tools. In practice, immersive VR systems produce event-based and kinematic data at frame-dependent rates, whereas wearable devices acquire physiological and motion signals at device-specific sampling frequencies. A clinically usable platform therefore requires a common time base and a middleware layer that timestamps all incoming signals and task events before fusion. From an implementation perspective, this can be achieved through packet-level timestamping, clock alignment at session start, and periodic drift correction during execution. Calibration procedures should include both hardware calibration and functional calibration. Hardware calibration involves checking sensor offsets, controller alignment, and communication latency. Functional calibration involves acquiring a short standardized baseline before each session, for example, neutral standing or sitting posture, resting heart rate, and a brief sequence of predefined reaching or stepping actions. This baseline provides a patient-specific reference for subsequent within-session adaptation and longitudinal comparisons. Future validation studies should not be limited to clinical outcomes alone but should also quantify core technical performance indicators of the integrated system. These include synchronization error between VR events and wearable streams, end-to-end latency, feature stability across repeated sessions, robustness to temporary signal degradation, and agreement between extracted digital metrics and conventional clinical outcome measures. Reporting these parameters will be essential to determine whether the proposed framework is not only conceptually sound but also technically reliable and suitable for clinical translation.

In addition, robust signal processing is necessary to make multimodal monitoring clinically reliable. Real-time pipelines should therefore include noise filtering, artifact handling, and signal-quality checks before feature extraction. This is especially relevant in home-based settings, where sensor placement, motion artifacts, transient connectivity issues, and environmental variability may reduce signal stability. For this reason, data fusion should rely on quality-controlled inputs and on feature extraction procedures that remain interpretable and sufficiently robust under non-ideal acquisition conditions.

### 2.2. Enhancing Engagement and Motivation Through Gamification

Gamification—the use of game design elements in non-game contexts—is a central strategy in our proposed model to make rehabilitation more motivating. In the context of VR telerehabilitation, gamification means structuring therapy exercises like a game: with goals, rules, challenges, and rewards that tap into the patient’s intrinsic and extrinsic motivation. This approach directly addresses the engagement/adherence challenge by turning therapy from a chore into a more enjoyable, even addictive, activity. Key gamification elements include point scoring; performance feedback; progress tracking (e.g., leveling up and achievement badges); timed challenges; leaderboards or competition; and narrative or theme to provide context. By introducing reward mechanisms and goal-setting in this way, rehabilitation exercises become more like interactive challenges to solve, which can sustain the patient’s interest and effort [6]. Importantly, gamified VR exercises can be tailored to the individual’s preferences—for instance, some patients might find a competitive game appealing while others respond more to collaborative or exploratory game styles.

There is mounting evidence that gamified rehabilitation interventions improve both patient engagement and clinical outcomes. Studies have demonstrated that incorporating game features into therapy can lead to greater repetitions of movement (practice dose) and improved motor function compared to standard exercises [6,10]. In stroke patients, for example, game-based training has been shown to induce significant gains in upper limb motor function and even promote neuroplastic changes, underlining the therapeutic potential of gamification [6]. A scoping review of gamified and digital rehab interventions found benefits not only in motor performance but also in cognitive function and overall therapy outcomes across conditions ranging from stroke to neurodegenerative diseases [6]. These improvements are mediated by increased motivation: patients stick with the program longer and perform more intensely when it feels like a game rather than an exercise prescription. Objective adherence data support this: remote programs that incorporated gamification and real-time feedback achieved completion rates on the order of 73–90%, substantially better than typical adherence rates for home exercise programs [11]. Gamification, therefore, addresses one of the weaknesses of rehabilitation, the human tendency to discontinue tedious tasks, making therapy fun, rewarding and goal-oriented.

Several psychological theories explain why gamification is effective in this context. According to Self-Determination Theory, supporting a person’s basic needs for autonomy, competence, and relatedness fosters intrinsic motivation. Well-designed rehabilitation games can satisfy these needs: patients feel autonomy when they choose how to play or see the impact of their own effort; they feel competence as they master challenges and receive positive feedback; and they feel relatedness through multiplayer or socially connected features like competing with others or encouragement from a remote coach avatar [6]. Likewise, Flow Theory suggests that people achieve a state of optimal engagement (“flow”) when facing tasks that are neither too easy nor too hard, with clear goals and immediate feedback. Gamified VR can dynamically adjust difficulty and provide instantaneous performance feedback (scores, visual cues), facilitating this flow state where patients are fully absorbed in the activity [6]. In rehabilitation terms, this means a patient might practice far longer and with higher quality of movement because they are immersed in the experience and intrinsically driven to succeed in the game.

Ultimately, by merging therapeutic goals with enjoyable gameplay, gamification helps ensure that patients not only adhere to their rehab programs more consistently but also perform their exercises with higher quality (since motivation and feedback improve concentration and effort). The result is a greater therapeutic dose and potentially better functional recovery in the long run, as suggested by early clinical studies and patient feedback on such systems [4,6].

From a clinical perspective, gamification should be viewed not as an entertainment layer but as a therapeutic strategy to increase dosage, consistency, and quality of practice.

### 2.3. Real-Time Monitoring and Adaptive Therapy

A cornerstone of the proposed VR-biosensor telerehabilitation model is its capacity for real-time monitoring and dynamic adaptation of therapy. Unlike traditional rehabilitation protocols that are relatively static (e.g., a fixed set of exercises at a set difficulty until the next clinical reassessment), a digitally integrated system can continually assess the patient’s performance and status during each session and automatically adjust the therapy parameters on the fly. This creates an adaptive therapy loop: sensing, analysis, and feedback operate in real time to personalize the intervention to the patient’s immediate needs.

At present, many adaptive mechanisms rely on rule-based systems, while more advanced AI-driven personalization remains an active area of research.

From a monitoring standpoint, the combination of VR interaction data and wearable sensor data yields a comprehensive view of the patient’s state. The system captures a range of objective metrics, for example, movement accuracy, speed, range of motion, number of errors, response times, heart rate, and perhaps even indicators of cognitive load or fatigue inferred from performance decrements. These metrics serve as digital biomarkers of the patient’s motor and cognitive function. Importantly, they can be tracked continuously and discreetly as part of the gameplay. In a closed-loop rehabilitation setting, the most relevant digital biomarkers are not abstract latent variables but clinically interpretable features extracted from multimodal task execution. These include motor biomarkers such as movement accuracy, range of motion, reaction time, reaching trajectory efficiency, postural sway, gait-related timing, and variability of task execution; cognitive biomarkers such as sustained and selective attention, visual working memory performance, planning efficiency, distractor management, and dual-task cost; and physiological biomarkers such as heart-rate response, respiratory rate, recovery trends, and abnormal stress-related patterns during exposure to immersive tasks. Within this framework, cognitive profiling is derived functionally from task performance rather than from direct neurophysiological acquisition, while physiological monitoring is primarily intended to support safety assessment and workload calibration.

These biomarkers should be validated against established clinical scales rather than being treated as standalone outcomes. The validation process should assess convergent validity, correlation with conventional rehabilitation endpoints, test-retest reliability, sensitivity to change, and feasibility in repeated home-based sessions. In other words, digital biomarkers become clinically meaningful only when they demonstrate a stable, interpretable, and clinically relevant relationship with standard rehabilitation measures.

Algorithmic bias is a major concern in rehabilitation AI because models are often trained on small, clinically heterogeneous, and demographically unbalanced datasets. To reduce this risk, model development should follow a conservative strategy: use stratified recruitment across diagnostic groups and severity levels; maintain balanced representation by age, sex, digital literacy, and functional status whenever feasible; prefer simpler and more interpretable models when data volume is limited; report subgroup performance explicitly; and continuously monitor model drift after deployment. Data augmentation and transfer learning may be useful in selected cases, but they should not substitute for representative clinical sampling. Equally important, the training dataset should include failed attempts, interrupted sessions, and high-variability performances rather than only successful task completions, because excluding these cases may bias the model toward fitter or more technology-confident users. For home-based rehabilitation, fairness also includes technological robustness: algorithms should remain reliable when signal quality is reduced by domestic noise, intermittent connectivity, or imperfect sensor placement. These safeguards are necessary to ensure that adaptive systems do not inadvertently amplify disparities in already vulnerable patient populations.

The moment-to-moment monitoring enables the system to detect meaningful changes or events. For instance, if a patient’s hand tremor increases or their movement speed slows, the system notes it; if their heart rate exceeds a predefined threshold during an exercise, that is registered; if they begin to consistently achieve perfect scores, indicating the task may be too easy, that is recognized as well. All this information feeds into the adaptation engine. This ensures an optimal challenge point, which is critical for motor learning and avoids plateaus. Another form of adaptation is adjusting assistive feedback: the system can provide more guidance when needed (such as visual cues or “virtual assistance” in movement trajectory if it senses the patient is deviating significantly) and fade out that assistance as the patient improves, thereby encouraging independent performance.

AI and machine learning can enhance these adaptive capabilities by learning from patient data over time. Machine learning models can analyze the patterns in a patient’s performance and identify subtle trends [6]. For instance, an AI algorithm might integrate multiple sensor inputs (motion smoothness, reaction time variability, and physiological signals) to determine the patient’s engagement level or physical exertion and then decide to insert a short rest break or switch to a different activity to prevent burnout. Over multiple sessions, machine learning could tailor a rehabilitation regimen specifically to the patient’s profile, emphasizing exercises that address their weaker areas and progressing at an optimal rate for that individual [6]. This kind of personalization is difficult to achieve with a fixed protocol or even with human therapists who see the patient only periodically, but a digital system “living” with the patient’s data continuously can fine-tune therapy in nuanced ways.

Additionally, real-time monitoring enables timely biofeedback to the patient. Biofeedback refers to making the patient aware of their internal physiological or performance metrics so they can self-correct. In our integrated VR scenario, a patient could be shown their own biometrics in an intuitive form, for example, a color change in the environment if their heart rate goes too high (signaling them to rest). In one study of remote sensor-based training for chronic pain, systems providing real-time movement biofeedback (like visualizing spine motion during exercises) helped patients improve movement quality and reduced pain more effectively than no-feedback exercises [11]. The combination of immediate feedback and adaptation essentially creates a responsive virtual therapist that continuously coaches the patient.

For the adaptive layer, a useful distinction should be made between interpretable rule-based adaptation and data-driven machine learning. In early-stage clinical deployment, rule-based algorithms are likely to be the safest and most transparent solution. For example, exercise duration, number of targets, distractor density, response-time thresholds, or task speed can be adjusted according to predefined performance and safety rules based on accuracy, completion time, movement smoothness, and physiological thresholds. Such logic is clinically auditable and facilitates therapist oversight.

As datasets grow, supervised learning models may be introduced to predict short-term outcomes such as fatigue, dropout risk, probability of successful task completion, or need for assistance. In practice, this would require labeled session-level data including task performance indicators, physiological responses, therapist annotations, support requirements, and interruption patterns. Interpretable supervised models are preferable in the initial translational phase, because they facilitate clinical review and help avoid opaque decision pathways. Reinforcement learning may become relevant only at a later stage, once sufficient safety constraints and clinically validated reward functions are available, because autonomous policy updates in fragile neurological populations raise important accountability issues. In this context, AI should not replace therapeutic decision-making but support it through human-in-the-loop recommendations that remain reviewable by the clinician.

To avoid black-box clinical decisions, every adaptive output should be traceable to observable variables and understandable system logic. Proposed adjustments should therefore be linked to interpretable features such as slower response times, increased error rate, reduced movement amplitude, postural instability, excessive cardiovascular load, or repeated need for assistance. This design principle is especially important in neurological rehabilitation, where therapist trust, patient safety, and medico-legal accountability are critical for adoption.

Because the proposed framework relies on continuous acquisition, transmission, and remote interpretation of sensitive behavioral and physiological data, data security and privacy compliance should be considered core implementation requirements rather than secondary technical details. End-to-end encryption, secure authentication procedures, role-based access control, and protected cloud or server infrastructures should be regarded as minimum requirements for clinically deployable systems. In addition, data governance policies should clearly define which data are collected, how they are processed, who can access them, and how long they are retained. These safeguards are especially important in home-based rehabilitation, where continuous monitoring may otherwise increase patient vulnerability and reduce trust in the system.

### 2.4. Home-Based Rehabilitation and Clinical Oversight

One of the greatest promises of telerehabilitation is bringing effective therapy into the patient’s home. A home-based approach removes many logistical barriers: travel to a clinic, scheduling constraints, and associated costs. Thereby facilitating more frequent and convenient therapy sessions. The VR and wearable biosensor telerehab model is inherently designed for home use, with the patient performing exercises in their living room or bedroom while immersed in a virtual environment.

In the home setting, the patient would typically have a VR headset and a set of wearable sensors (and possibly small accessories like a balance board or instrumented objects) provided by the clinic or healthcare system. These devices today are increasingly user-friendly: standalone VR headsets are now wireless and relatively simple to operate, and sensors can sync automatically to a smartphone or computer application. The rehabilitation program (which games or scenarios to do, for how long, and at what difficulty) can be configured remotely by the clinician and updated as needed.

Throughout each home session, clinical oversight is maintained remotely via data connectivity. The system can stream or periodically upload the patient’s performance data to a secure cloud platform that the clinician accesses. In some implementations, therapists can even monitor the session live. One recent telerehab platform (“RecoveryFun”) for post-stroke upper limb rehab consists of a VR headset with a wearable motion sensor and is paired with a caregiver mobile app and a clinician web portal. In a pilot deployment, physiotherapists were able to remotely monitor patients’ exercise performances in real time, adjusting the difficulty and number of exercise repetitions through the platform’s interface, all while considering the patient’s fatigue and stress levels as indicated by the system [2]. This kind of real-time telemonitoring means the therapist can virtually “peek” into the session and intervene if needed.

Even when live supervision is not occurring, the data logs enable asynchronous oversight. Therapists can review summary reports of each session, including metrics like exercise completion, scores, range of motion achieved, error counts, and heart rate trends through the clinician dashboard. This allows them to keep track of patient progress on a regular basis (daily or weekly) rather than waiting for the next clinic visit. This timely insight can lead to earlier interventions and better outcomes [12,13]. The ability for therapists to have continuous remote visibility into the patient’s rehab is a game-changer, effectively extending professional care into the home environment. Moreover, patients often report feeling reassured knowing that their therapist is “connected” and watching over their progress, even if not physically present.

The scalability of home-based immersive rehabilitation depends not only on clinical efficacy but also on cost, usability, digital inclusion, and implementation feasibility. This is particularly relevant for older adults and patients with neurological impairment, who may have reduced familiarity with digital devices, motor limitations affecting setup, or limited access to stable broadband connections. A realistic deployment model should therefore avoid assuming a high-end, technology-rich home environment. Several mitigation strategies can reduce this barrier. First, the hardware configuration should be modular, with a minimum viable setup based on a standalone headset and one or two low-burden wearable sensors, while more complex peripherals are reserved for clinic-based or advanced cases. Second, the onboarding process should include guided installation, caregiver-assisted setup when needed, short familiarization sessions, and simplified interfaces with large visual cues, low text density, and step-by-step prompts. Third, platforms should support asynchronous data upload and offline-first task execution whenever possible so that temporary connectivity limitations do not prevent therapy delivery. Fourth, a hybrid care model may be preferable, in which initial calibration and training are performed in the clinic and only then transitioned to the home setting once the patient demonstrates adequate safety and usability.

From a health-system perspective, scalability also requires service-level solutions. Device loan programs, shared-equipment pathways, centralized technical support, and therapist dashboards that reduce monitoring burden can improve sustainability. In this sense, accessibility should not be framed as a secondary implementation issue but as a core design constraint of the bioengineering framework itself.

Home-based VR telerehabilitation has been tested in various patient populations and shown promising results in terms of both usability and efficacy. Overall, the ability to engage in rehabilitation without leaving home led to greater therapy adherence and reduced burden on caregivers, as the patients did not have to travel and could perform exercises at convenient times [2]. As one might expect, home rehab can particularly benefit those with mobility limitations or in rural areas, but even for others it offers convenience and the potential for more frequent, shorter sessions that fit daily life.

From the clinician’s perspective, home-based telerehab demands new workflows. Therapists transition from physically guiding exercises to analyzing data and guiding remotely. They might spend part of their day reviewing patient dashboards and sending feedback or modifying virtual therapy “prescriptions”. This can actually be efficient: one therapist could supervise multiple patients remotely in a time-sliced manner, given that not every session needs real-time attention. It also allows specialists to reach patients beyond their immediate geographic area. A challenge is ensuring that clinicians are trained to use these digital tools and interpret the data. As the paradigm gains acceptance, rehabilitation professionals are increasingly recognizing the value of these skills. At the same time, this model requires careful consideration of clinician workload, data interpretation demands, and the need for adequate training in digital rehabilitation tools.

In summary, home-based immersive telerehabilitation provides a pathway to high-intensity, patient-centered therapy at home with continuous clinical oversight. It blends the convenience and comfort of being at home with the structure and supervision of clinic-based therapy [14]. By keeping therapists connected to the patient’s daily rehabilitation and leveraging technology to fill the gap of physical distance, the home-based model can maintain a high standard of care. It exemplifies the broader digital transformation of rehabilitation: shifting appropriate aspects of therapy from hospital to home while preserving personalization and human oversight through connectivity.

### 2.5. System Architecture and Control Model

**Figure 1:**
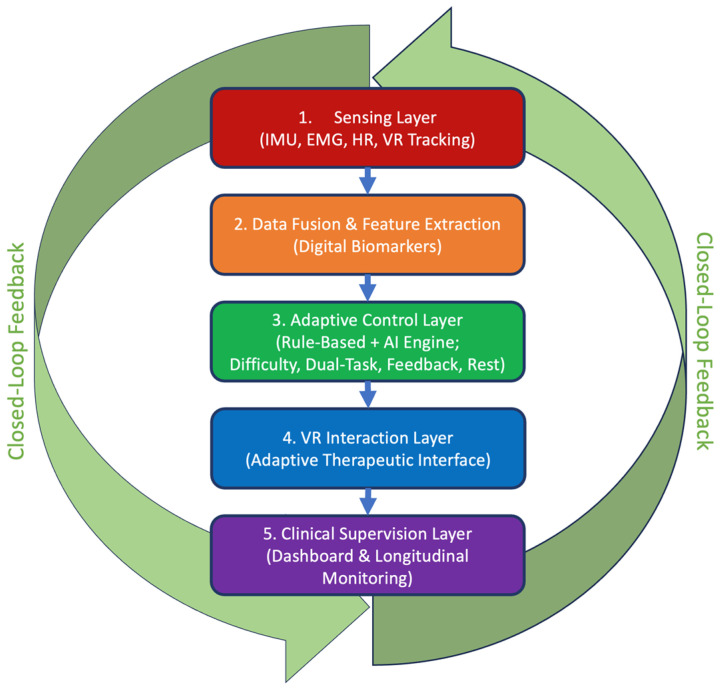
Block-diagram representation of the proposed five-layer closed-loop VR telerehabilitation architecture, illustrating the flow from multimodal sensing and digital biomarker computation to adaptive control, VR environment modulation, and remote clinical supervision via a longitudinal monitoring dashboard.

To move beyond a conceptual narrative and toward an engineering-grade telerehabilitation paradigm, we propose a structured closed-loop system architecture integrating immersive virtual reality (VR), multimodal biosensing, adaptive control, and clinical supervision. The architecture is organized into five functional layers operating within a continuous feedback loop (Figure 1).

#### 2.5.1. Layered System Architecture

The proposed framework comprises:

(1) Sensing Layer

This layer acquires multimodal patient data during VR interaction. Inputs include:

• Kinematic data (joint angles, velocity, acceleration) from IMUs and VR tracking;
• Surface EMG signals (muscle activation patterns);
• Cardiovascular metrics (heart rate, HRV-derived parameters);
• Performance indicators (reaction time, error rate, task completion time);
• All signals are time-synchronized and streamed to the data fusion module.

(2) Data Fusion and Feature Extraction Layer

Raw signals are preprocessed using filtering and normalization pipelines. Extracted features may include:

• Movement smoothness indices (e.g., spectral arc length);
• Variability metrics (coefficient of variation of reaction times);
• Fatigue indicators (RMSSD, LF/HF ratio from HRV);
• Accuracy and error probability distributions.

Let the multimodal feature vector at time t be defined as:

$$X_{t}=[K_{t},M_{t},P_{t},C_{t}]$$

where

• $K_{t}$ = kinematic feature set;
• $M_{t}$ = muscular activation features;
• $P_{t}$ = physiological metrics;
• $C_{t}$ = cognitive-performance indicators.

This vector constitutes the digital biomarker state of the patient at time t.

(3) Adaptive Control Layer

The adaptive engine dynamically regulates therapy parameters based on patient state. Control variables include:

• Task difficulty level;
• Stimulus density;
• Dual-task complexity;
• Feedback intensity;
• Rest intervals.

The system implements a closed-loop adaptive rule:

$$D_{t+1}=D_{t}+\alpha \cdot (S_{t}-S_{target})-\beta \cdot F_{t}$$

where

• $D_{t}$ = current difficulty level;
• $S_{t}$ = composite performance score;
• $S_{target}$ = optimal challenge threshold;
• $F_{t}$ = fatigue index derived from physiological signals;
• α,β = adaptive gain parameters.

The composite performance score is defined as:

$$S_{t}=w_{1}\cdot A_{t}+w_{2}\cdot V_{t}-w_{3}\cdot E_{t}$$

where:

• $A_{t}$ = task accuracy;
• $V_{t}$ = movement velocity efficiency;
• $E_{t}$ = error rate;
• $w_{i}$ = weighting coefficients.

This ensures that task progression maintains the patient within an optimal learning zone, consistent with motor learning theory and flow-based engagement models.

(4) VR Interaction Layer

The VR engine translates control outputs into environmental modifications:

• Increased cognitive load (additional stimuli);
• Narrower movement tolerance windows;
• Reduced visual cues;
• Time constraints.

The environment therefore acts as an adaptive therapeutic interface governed by physiological and performance-driven control signals.

(5) Clinical Supervision Layer

Aggregated session-level metrics are transmitted to a secure clinical dashboard. Clinicians access:

• Longitudinal performance trends;
• Fatigue-risk alerts;
• Adherence statistics;
• Digital biomarker trajectories.

Let:

$$Z_{session}=\sum_{t=1}^{T}X_{t}$$

represent the integrated session-level digital phenotype used for remote assessment.

#### 2.5.2. Artificial Intelligence-Enhanced Personalization

Beyond rule-based adaptation, machine learning models can refine personalization. At this stage, the following formulations should be interpreted as illustrative control and personalization models for future development, rather than as already validated deployment-ready algorithms. A supervised learning model may estimate recovery trajectory:

$${\hat{R}}_{t+k}=f(X_{1:t})$$

where ${\hat{R}}_{t+k}$ predicts functional improvement at future time t+k.

Alternatively, reinforcement learning can optimize difficulty progression by maximizing a reward function:

$$R=\lambda_{1}\cdot \Delta Performance-\lambda_{2}\cdot Fatigue-\lambda_{3}\cdot ErrorPersistence$$

The policy π selects the next therapeutic action:

$$a_{t+1}=\pi (X_{t})$$

This transforms telerehabilitation into a dynamic optimization problem rather than a static protocol.

#### 2.5.3. Digital Biomarkers and Standardization

A key engineering contribution of this framework is the operationalization of multimodal digital biomarkers. These include:

• Motor efficiency index;
• Cognitive-motor integration score;
• Fatigue-resilience metric;
• Adaptive progression slope.

Standardizing these indices may facilitate cross-platform comparability and multicenter validation.

#### 2.5.4. Closed-Loop Paradigm Summary

The proposed system can be summarized as:

Patient State→Feature Extraction→Adaptive Control→VR Modification→Updated Patient State

This continuous loop enables:

• Real-time personalization;
• Safety monitoring;
• Quantitative progress tracking;
• Scalable remote supervision.

In contrast to conventional telerehabilitation models, which often operate in an open-loop manner (fixed protocols with delayed reassessment), this bioengineering-driven architecture establishes a responsive and data-centric therapeutic ecosystem suitable for home-based neurorehabilitation.

## 3. Future Directions and Research Roadmap

Although the present article builds on a growing body of literature supporting immersive VR, wearable monitoring, and telerehabilitation, the current evidence base remains methodologically heterogeneous. Existing studies differ substantially in sample size, intervention duration, target population, technological configuration, and outcome measures, and many are based on pilot, feasibility, or proof-of-concept designs rather than large-scale controlled validation. Moreover, the integrated combination of immersive VR, wearable biosensing, adaptive feedback, and home-based deployment has not yet been validated in a standardized way across neurological populations. For this reason, the proposed framework should not be interpreted as already clinically validated in its fully integrated form, but rather as a structured translational model informed by converging, yet still incomplete, evidence. Future work should therefore move beyond proof-of-concept enthusiasm and address comparative validation, implementation robustness, long-term usability, and real-world clinical applicability.

Future research should prioritize (i) large-scale clinical validation, (ii) standardization of digital outcome measures, and (iii) integration of VR-based telerehabilitation into existing healthcare pathways.

While the integration of immersive VR and wearable biosensors into telerehabilitation is highly promising, significant work remains to be done to validate and refine this model. In this section, we highlight key future directions and research priorities that will pave the way for translating this conceptual paradigm into standard practice.

Rigorous clinical trials remain a priority to validate the efficacy, safety, and generalizability of VR-based telerehabilitation [9]. Larger, multicenter randomized studies with diverse patient populations and standardized protocols are needed to establish robust evidence and inform clinical guidelines. Parallel research on health economics is essential to determine the cost-effectiveness and scalability of VR-based telerehabilitation [14]. Demonstrating favorable economic outcomes, such as reduced healthcare utilization and improved access, will be vital for broad adoption and reimbursement in healthcare systems. Continued technological innovation is required to enhance VR telerehabilitation platforms. Future efforts should focus on improving hardware comfort and immersion, advancing software (e.g., adaptive algorithms and AI-driven feedback), and ensuring interoperability with telehealth systems to provide more reliable and scalable interventions. User experience is another critical focus for future research. Systems should be co-designed with patients and clinicians and undergo rigorous usability testing to maximize acceptability, accessibility, and patient engagement. Ethical and legal frameworks must also evolve alongside these innovations [6,15]. Future work should address data privacy and security, patient safety in virtual environments, and equity of access to prevent a digital divide in VR-based care. Finally, the scope of VR-based telerehabilitation should be expanded across a broader range of conditions and settings. Research should explore its application in diverse rehabilitation domains and populations (including different ages and impairments) and how to integrate these services into existing healthcare pathways on a larger scale.

In summary, the future research agenda is broad and exciting. It spans clinical validation, technical innovation, user-centered design, and system-level implementation studies. Immersive VR combined with wearable biosensors represents a bold reimagining of rehabilitation, but to realize its full potential, stakeholders must ensure it is effective, safe, equitable, and seamlessly integrated into healthcare. By systematically investigating the areas outlined above, researchers and clinicians will build the evidence and frameworks required to move this field from experimental pilots to standard practice. The ultimate vision is that in a decade’s time, a stroke survivor or a person with Parkinson’s might routinely receive, as part of their care, a personalized home VR rehab kit monitored by experts, making rehabilitation not only effective but also engaging and accessible to all who need it.

## 4. Conclusions

Telerehabilitation is undergoing a meaningful transformation through the convergence of immersive virtual reality and wearable biosensor technologies. This perspective has outlined how rehabilitation can evolve toward home-based, engaging, and data-driven models that extend care beyond clinical settings while maintaining therapeutic rigor. By embedding structured exercises within immersive VR environments and integrating real-time biosensor monitoring, rehabilitation can become more interactive, personalized, and responsive to patient performance. Gamification strategies support sustained motivation and adherence, while adaptive feedback mechanisms allow therapy intensity and complexity to be dynamically adjusted. Together, these elements create a closed-loop rehabilitation framework that integrates motor, cognitive, and physiological dimensions.

Rather than presenting this integration as an already established solution, the present article proposes a translational bioengineering roadmap that identifies the main technical, clinical, and implementation requirements for future development. Future research should evaluate not only efficacy but also synchronization robustness, signal quality management, biomarker validity, algorithmic transparency, privacy protection, and real-world accessibility.

## Data Availability

No new data were created or analyzed in this study..
